# Impact of two early treatment protocols for anterior dental crossbite on children’s quality of life

**DOI:** 10.1590/2177-6709.23.1.071-078.oar

**Published:** 2018

**Authors:** Cristina Batista Miamoto, Leandro Silva Marques, Lucas Guimarães Abreu, Saul Martins Paiva

**Affiliations:** 1 Universidade Federal de Minas Gerais, Departamento de Odontopediatria e Ortodontia (Belo Horizonte/MG, Brazil).; 2 Universidade Federal dos Vales do Jequitinhonha e Mucuri, Departamento de Odontopediatria e Ortodontia (Diamantina/MG, Brazil).

**Keywords:** Children, Malocclusion, Anterior crossbite, Interceptive orthodontics, Quality of life

## Abstract

**Objective:**

To assess the impact of two early treatment protocols for anterior dental crossbite on children’s quality of life.

**Methods:**

Thirty children, 8 to 10 years of age, with anterior dental crossbite, participated in this study. Individuals were divided into two groups: Group 1 - 15 children undergoing treatment with an upper removable appliance with digital springs; Group 2 - 15 children undergoing treatment with resin-reinforced glass ionomer cement bite pads on the lower first molars. Quality of life was evaluated using the Brazilian version of the *Child Perceptions Questionnaire* (CPQ_8-10_), which contains four subscales: oral symptoms (OS), functional limitations (FL), emotional well-being (EW), and social well-being (SW). A higher score denotes a greater negative impact on children’s quality of life. Children answered the questionnaire before treatment (T_1_) and twelve months after orthodontic treatment onset (T_2_). Descriptive statistics, the Wilcoxon test and analysis of covariance (ANCOVA) were performed.

**Results:**

Children’s mean age was 9.07 ± 0.79 years in Group 1 and 9.00 ± 0.84 years in Group 2. For Group 1, the FL and EW subscale scores and the overall CPQ_8-10_ were significantly higher in T_1_ as compared to T_2_ (*p*= 0.004, *p*= 0.012 and *p*= 0.015, respectively). For Group 2, there were no statistically significant differences. The ANCOVA showed no significant difference regarding quality of life at T_2_ between groups, after controlling for quality of life measures at T_1_.

**Conclusions:**

The difference regarding the impact on quality of life between groups is not related to the protocol used.

## RESUMO

**Objetivo:**

avaliar o impacto de dois protocolos de tratamento precoce para a mordida cruzada anterior dentária na qualidade de vida de crianças.

**Métodos:**

trinta crianças de 8 a 10 anos de idade com mordida cruzada anterior dentária participaram desse estudo. Os indivíduos foram divididos em dois grupos: Grupo 1 - 15 crianças em tratamento com aparelho removível superior com molas digitais; Grupo 2 - 15 crianças em tratamento com batentes de cimento de ionômero de vidro resinoso nos primeiros molares permanentes inferiores. A qualidade de vida foi avaliada por meio da versão brasileira do *Child Perceptions Questionnaire* (CPQ_8-10_), o qual contém quatro subescalas: sintomas bucais (SB), limitações funcionais (LF), bem-estar emocional (BE) e bem-estar social (BS). Um escore mais alto indica um impacto mais negativo na qualidade de vida. As crianças responderam ao questionário antes do tratamento (T_1_) e 12 meses após o início do tratamento ortodôntico (T_2_). Estatística descritiva, o teste Wilcoxon e análise de covariância (ANCOVA) foram realizados.

**Resultados:**

a média de idade das crianças foi de 9,07 ± 0,79 anos no Grupo 1 e de 9,00 ± 0,84 no Grupo 2. Para o Grupo 1, os escores das subescalas LF e BE e o escore total do CPQ_8-10_ foram significativamente maiores em T_1_ do que em T_2_ (*p*= 0,004, *p*= 0,012 e *p*= 0,015, respectivamente). Para o Grupo 2, não houve diferença estatisticamente significativa. A ANCOVA não mostrou diferença significativa entre os grupos em relação à qualidade de vida em T_2_, após o controle para as medidas de qualidade de vida em T_1_.

**Conclusões:**

a diferença em relação ao impacto na qualidade de vida entre os grupos não foi relacionada ao protocolo de tratamento utilizado.

## INTRODUCTION

The concept of oral health-related quality of life (OHRQoL) has been used to measure the impact of oral outcomes on the functions and quality of life of individuals.^1^ Recently, one of the objectives of dental research has been to assess the OHRQoL of children and adolescents, since oral diseases, such as dental caries and malocclusion, have a negative effect on the physical and psychological well-being of young people.^2^ ^,^ ^3^ Generally, the instruments used to assess OHRQoL are constructed in the form of surveys consisting of questions aimed at measuring how much oral outcomes affect people’s lives and daily routines by means of responses organized in numerical scales.^4^

Anterior dental crossbite occurs when there is a change in the inclination of one or more anterior teeth with the upper incisor(s) positioned palatally in relation to the lingual surface of the lower teeth.^5^ Studies evaluating these changes reported the possibility of periodontal problems in the lower incisors, the presence of discomfort, alteration in the anteroposterior position of the mandible, and problems with the temporomandibular joint (TMJ), when the problem is not treated early.^6^ ^,^ ^7^ Interceptive orthodontic intervention in the mixed dentition allows the orthodontist to correct the anterior crossbite earlier in a way that promotes the harmonious growth of the bone bases,^8,9^ mitigating the chances of severe disorders in the permanent dentition.

The impact of orthodontic treatment with fixed appliances on the quality of life of children and adolescents has been explored in depth in prior literature.^10^ ^,^ ^11^ However, the association between interceptive orthodontic treatment and OHRQoL still needs to be properly investigated.^12^ ^,^ ^13^ It is important to consider relevant aspects of the patient’s quality of life during orthodontic treatment, such as potential psychosocial problems and functional disabilities caused by the wearing of orthodontic devices.^14^ Therefore, the objective of this study was to assess the impact of two early treatment protocols for correction of the anterior dental crossbite (upper removable appliance with digital springs; and resin-reinforced glass ionomer cement bite pads on the lower first molars) on the quality of life of children. The null hypothesis was that there is no difference between both protocols regarding the impact on children’s quality of life.

## METHODS

### Participants, study site, and eligibility criteria

The sample of this prospective study was selected from the registry of patients attending the Children’s Clinic of the Federal University of the Valleys of Jequitinhonha and Mucuri (UFVJM), located in the city of Diamantina, Brazil. The study was conducted between March, 2014 and December, 2015. Individuals between 8 and 10 years of age and who presented anterior dental crossbite in the mixed dentition, with the presence of the four first permanent molars and at least one crossed permanent incisor, were included in this study. Exclusion criteria were: (I) impairment in general health based on medical history and physical examination, (II) anterior skeletal or functional crossbite, (III) posterior crossbite associated with anterior crossbite, (IV) presence of sucking habits or individuals who had stopped the habit less than a year before the study’s onset, (V) previous history of orthodontic treatment, and (VI) individuals with any oral disease or those who had undergone any kind of dental treatment within the last month.

The sample consisted of 30 individuals, 8 to 10 years of age, with anterior dental crossbite in the mixed dentition. The participants were divided into two groups: Group 1 consisted of 15 patients undergoing treatment with an upper removable appliance with digital springs; Group 2 consisted of 15 patients undergoing treatment with resin-reinforced glass ionomer cement bite pads on the lower first molars. The distribution of 30 individuals between the two groups was performed randomly as follows: an envelope was prepared with 30 records with the names of the two treatment protocols, each mode containing 15 records. A card was selected from the envelope for each participant, indicating the group to which he/she belonged. This process was carried out by an assistant. 

### Ethical considerations

The research proposal was submitted to and approved by the Ethics Committee on Human Research from UFVJM (protocol #525.056). Children and their guardians were informed about the study and that their participation was entirely voluntary. The children who agreed to participate in the study signed an informed consent form, as did their parents or guardians. After the follow-up period, patients whose anterior dental crossbite had not been fully corrected continued the treatment or were subjected to a new type of therapy.

### OHRQoL assessment tool

Participants’ OHRQoL was assessed by means of the Brazilian version of the Child Perceptions Questionnaire (CPQ_8-10_), which is a tool used to assess the impact of oral conditions on the quality of life of children from 8 to 10 years of age.^15^ The CPQ_8-10_ consists of 25 questions divided into four subscales: oral symptoms (OS), with 5 questions; functional limitations (FL), with 5 questions; emotional well-being (EW), with 5 questions; and social well-being (SW), with 10 questions. An ordinal scale provides the following response options for each question: never (0), once/twice (1), sometimes (2), often (3), and every day/almost every day (4). The scores for each subscale are computed by adding up the scores for each question. The overall score is calculated by adding the scores of the four subscales. The overall score ranges from 0 to 100 points. Higher values indicate a more negative impact of the oral outcome on children’s quality of life. The CPQ_8-10_ was translated and adapted cross-culturally for the Brazilian population with similar psychometric properties to the original version.^16^

Data were collected through surveys that were answered in an average time of 15 minutes in a separate room next door to the clinic. The subjects answered them on two occasions: the first occurred before placing the two types of protocols, for determining the baseline (T_1_); the second assessment was carried out twelve months after the onset of the interceptive orthodontic treatment (T_2_). Treatments were conducted by a specialist in orthodontics , who stressed the benefit of the early treatment of anterior dental crossbite for the children and their parents/caregivers. Shortly after the placement of the resin-reinforced glass ionomer cement bite pads or the upper removable appliance with digital springs, the participants and their parents/caregivers were given instruction on diet restrictions, hygiene, and the commitment required by orthodontic treatment. This information was emphasized again on subsequent monthly appointments. Parents/caregivers were asked to check their own commitments before scheduling consultations for their children, to avoid delays or missing the consultations. A telephone number was provided in case of need for emergency consultation due to breakage or loss of devices.

### Early orthodontic protocols for anterior dental crossbite

#### Upper removable appliance with digital springs

**Figure 1:**
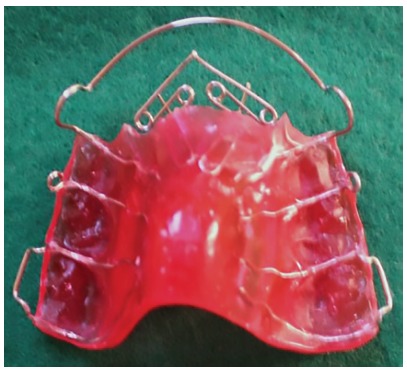
The upper removable appliance with digital springs.

The device presented two Adams clasps in the permanent upper first molars, two arrow clasps between the deciduous upper molars and a double spring adapted to the palatal surfaces of the teeth to be uncrossed, in addition to the vestibular arch (Fig 1). The posterior region presented an occlusal splint in an attempt to promote sufficient disocclusion, enabling the movement of the crossed teeth. The patients were advised to remove the appliance only to eat and during oral hygiene.

#### Resin-reinforced glass ionomer cement bite pads

**Figure 2:**
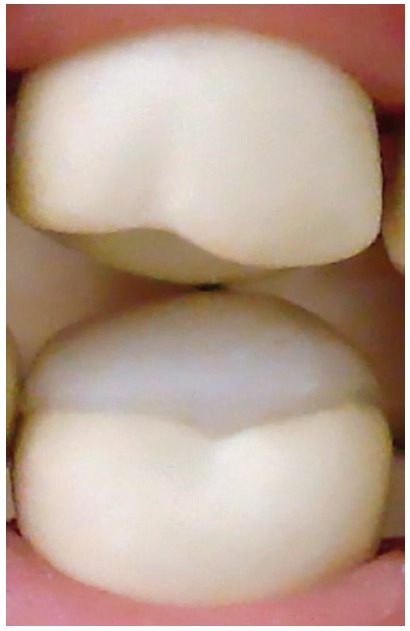
Glass ionomer cement bite pads

Resin-reinforced glass ionomer cement bite pads (Riva Light Cure^®^, Bayswater, Australia) were placed on the occlusal surface of the permanent lower first molars (Fig 2). These devices contained dimensions that were sufficient to promote the disocclusion of all of the anterior teeth, which allowed enough space for the movement of the crossed teeth by tongue pressure.

### Sample power calculation

The sample power calculation was carried out by means of the Power and Sample Size Calculation Program (PS, version 3.0, Nashville, Tenn, USA). The overall CPQ_8-10_ score was the outcome with which the sample power was calculated. For Group 1, the mean difference in the response of matched pairs was 15.53 and the standard deviation of this mean difference was 11.98. For Group 2, the mean difference in the response of matched pairs was 11.73 and the standard deviation of this mean difference was 10.68. The type I error was 0.05. Thus, the power of the study to identify significant differences between T_1_ and T_2_ was 99.4% for Group 1 and 97.3% for Group 2.

### Orthodontic treatment need assessment

Children’s orthodontic treatment need was assessed using the Dental Aesthetic Index (DAI). The DAI consists of scores for 10 occlusal characteristics. The score of each occlusal characteristic is multiplied by a linear regression coefficient and added together to the constant value of 13, resulting in the DAI final score. Based on DAI cut-offs, the children were assigned to four groups: slight need of treatment (DAI ≤ 25); elective treatment (DAI = 26 to 30); highly desirable treatment (DAI = 31 to 35), and mandatory treatment (DAI ≥ 36).^17^ Before study’s commencement, a training and calibration exercise guaranteed accuracy for the use of DAI.

### Monthly family income

The monthly family income was categorized in terms of the Brazilian minimum wage (BZMW), which was R$788.00 at the time of the study and was established as the sum of monthly income of all economically active members of that family. The children were then categorized as follows: those whose families had a monthly income of ≤ 1 BZMW; > 1 BZMW and ≤ 2 BZMWs; > 2 BZMWs and ≤ 5 BZMWs and those whose families had a monthly income of > 5 BZMWs and ≤ 10 BZMWs.

### Data analysis

Data from both groups were analyzed using the *Statistical Package for Social Sciences* (SPSS for Windows, version 20.0; SPSS Inc., Chicago, IL, USA). Descriptive statistics were calculated with the aim of providing the sample characteristics. The Shapiro-Wilk test was used to determine the distribution of data and the result showed that the data had non-normal distribution. Inter-group comparisons regarding children’s sociodemographic characteristics, orthodontic treatment need and CPQ_8-10_ scores at T_1_ were carried out using the Chi-square test and the Mann-Whitney test. 

The Wilcoxon signed-rank test was used to assess statistical differences between T_1_ and T_2_ for the subscales and overall CPQ_8-10_ score. For the overall score, the level of statistical significance was *p*< 0.05. The Bonferroni correction was used with the subscales for which the level of statistical significance was *p*< 0.013.

Evaluation of the relationship between the type of treatment protocol and the OHRQoL scores at T_2_, controlling for confounding variables was carried out by means of the analysis of covariance (ANCOVA). Confounding variables with a *p*< 0.20 in the inter-group comparisons were incorporated in the model. Again, the level of significance for the overall score was *p*< 0.05 and for the subscales, a *p*< 0.013 was considered statistically significant.

## RESULTS

Of the 15 children in Group 1, 11 were male (73.3%) and 4 were female (26.7%); the mean age of these children was 9.07 ± 0.79 years. Of the 15 children in Group 2, 7 were male (46.7%) and 8 were female (53.3%); the mean age was 9.00 ± 0.84 years. The socio-demographic characteristics of the participants are presented in Table 1. 

**Table 1:** Children’s sociodemographic characteristics, orthodontic treatment needs and quality of life before treatment.

	Group 1	Group 2	Intergroup comparison (p value)
Gender of the children			
Boys	11 (73.3)	07 (46.7)	0.264*
Girls	04 (26.7)	08 (53.3)	
Age of the children (years)			
8	04 (26.6)	05 (33.3)	0.999**
9	06 (40.0)	05 (33.3)	
10	05 (33.4)	05 (33.4)	
Orthodontic treatment needs			
Slight	01 (06.7)	02 (13.3)	0.999**
Elective	05 (33.3)	02 (13.3)	
Highly desirable	04 (26.7)	08 (53.4)	
Mandatory	05 (33.3)	03 (20.0)	
Family income (BZMW)			
Up to 1 BZMW	01 (06.7)	01 (06.7)	0.412**
From 1 to 2 BZMWs	07 (46.7)	10 (66.7)	
From 2 to 5 BZMWs	06 (40.0)	04 (26.7)	
From 5 to 10 BZMWs	01 (06.7)	00 (00.0)	
CPQ_8-10_ (T_1_)			
OS^1^	7.47 (7.00)^2^	4.40 (3.00)^2^	0.067***
FL^1^	6.13 (7.00)^2^	2.40 (1.00)^2^	0.011***
EW^1^	6.33 (6.00)^2^	2.40 (0.00)^2^	0.019***
SW^1^	7.80 (6.00)^2^	2.33 (1.00)^2^	0.032***
OL^1^	27.87 (35.0)^2^	11.13 (7.00)^2^	0.008***

BZMW = Brazilian monthly wage.

CPQ_8-10_ = Child Perceptions Questionnaire. T_1_ = before beginning treatment.

OS = oral symptoms; FL = functional limitations; EW = emotional well-being; SW = social well-being; OL = overall score.

^1^ Analyzed as a continuous variable. ^2^ Mean (Median). *Pearson chi-square. **Linear by linear chi-square. ***Mann-Whitney test.

Group 1 scores for the FL and EW subscales and the overall CPQ_8-10_ score were significantly higher in T_1_ as compared to T_2_ (*p*= 0.004, *p*= 0.012, and *p*= 0.015, respectively). There were no significant statistical differences in Group 2 (Table 2).

**Table 2:** Comparison of the medians and modes of CPQ_8-10_ subscale and overall scores for the two early treatment protocols of anterior dental crossbite.

	CPQ_8-10_ Variation	Median T_1_	Mode T_1_	Median T_2_	Mode T_2_	p value T_1_ - T_2_
Group 1						
OS	0 - 20	7.00	12	4.00	6	0.032*
FL	0 - 20	7.00	8	1.00	0	0.004*
EW	0 - 20	6.00	0	1.00	0	0.012*
SW	0 - 40	6.00	0	4.00	0	0.269*
OL	0 - 100	35.00	36	12.00	3	0.015**
Group 2						
OS	0 - 20	3.00	2	5.00	8	0.441*
FL	0 - 20	1.00	0	2.00	0	0.590*
EW	0 - 20	0.00	0	3.00	0	0.683*
SW	0 - 40	1.00	0	1.00	0	0.570*
OL	0 - 100	7.00	2	8.00	0	0.589**

CPQ_8-10_ = Child Perceptions Questionnaire.

T_1_ = before beginning treatment. T_2_ = 12 months after beginning treatment.

OS = oral symptoms; FL = functional limitations; EW = emotional well-being; SW = social well-being; OL = overall score.

* Wilcoxon signed-rank test and Bonferroni correction. Significance level < 0.013.

** Wilcoxon signed-rank test. Significance level < 0.05.

The results of the ANCOVA showed no significant difference in the subscale and CPQ_8-10_ overall scores at T_2_ between the two types of treatment protocol (Group 1 and Group 2), after controlling the model for children’s measures of quality of life at T_1_ (Table 3). 

**Table 3:** ANCOVA models demonstrating contribution of covariates to overall and subscale CPQ_8-10_ scores at T_2_.

	OS		FL		EW		SW		OL	
	F statistics	p value*	F statistics	p value*	F statistics	p value*	F statistics	p value*	F statistics	p value**
CPQ_8-10_ (T_1_)	1.5	0.231	7.3	0.012	7.24	0.012	17.98	0.001	7.49	0.011
Treatment protocol	0.96	0.335	0.02	0.9	0.65	0.424	0.36	0.553	0.76	0.39

CPQ_8-10_ = Child Perceptions Questionnaire.

T_1_ = before beginning treatment; T_2_ = 12 months after beginning treatment.

OS = oral symptoms; FL = functional limitations; EW = emotional well-being; SW = social well-being; OL = overall score.

*Significance level < 0.013. **Significance level < 0.05.

## DISCUSSION

The results of this study confirmed that no statistical difference was found in the OS, FL, EW and SW subscales as well as in the overall CPQ_8-10_ score between children wearing an upper removable appliance with digital springs and children who were treated with resin-reinforced glass ionomer cement bite pads on the lower first molars. The covariate that contributed most for individuals’ OHRQoL at T_2_ was the measures of quality of life at T_1_ in the FL, EW and SW subscales as well as in the overall CPQ_8-10_ score. 

 Within interceptive orthodontics, recent studies have shown the positive effect of orthodontic therapy on the OHRQoL of treated patients.^12^ ^,^ ^18^ It is important to understand that an improved function is not the only reason why many individuals seek treatment.^19^ ^,^ ^20^ The effects of malocclusion on emotional and social well-being are also important justifications for seeking orthodontic treatment,^21^ and these are the motivations that subjective indices, such as the CPQ_8-10_, also assess. OHRQoL has been considered a multidimensional construct, in regards to the frequency of the impact that oral conditions may have on physical aspects, such as oral symptoms and functional limitations. This construct also concerns the effects of oral outcomes on individuals’ psychosocial aspects.^22^ It has been recognized that malocclusion has a negative impact on children’s and adolescents’ quality of life, mostly on emotional and social well-beings^23^ and orthodontic treatment, on the other hand, improves OHRQoL with positive repercussions on functioning^18^ and self-esteem.^12^

OHRQoL assessment becomes relevant in the participants’ age group, especially with regard to anterior dental crossbite, given that correction in mixed dentition are recommended to avoid compromising the dentofacial condition, which could result in the development of periodontal issues due to traumatic occlusion^24^ or a skeletal Class III malocclusion.^8^ ^,^ ^9^ These findings may indicate the need for the orthodontist to prioritize the early correction of this irregularity^25^ and any other irregularities, such as the presence of crowding in the anterior region,^26^ to improve the patients’ perception in regard to their dental appearance. This work highlights the importance of diagnosis and early intervention for anterior dental crossbite using orthodontic devices that seem to correct this malocclusion quickly and effectively, with minimal discomfort to the child.^27^

This study has limitations that need to be acknowledged. The first regards the income of the families. Most participants belonged to families with monthly income of less than 5 BZMWs. The present evaluation would have benefited of a more equalized sample in terms of socioeconomic characteristics. Although some factors that could influence the results were controlled, such as having treatments performed by only one practitioner, other factors were not controlled, such as differences (intragroups) regarding the severity of malocclusion.^28^ Moreover, children in the second assessment did not demonstrate the same stage of correction in their anterior dental crossbite. 

A systematic review^29^ on the treatment of anterior dental crossbite showed that most of the articles published about therapy protocols for this malocclusion are case reports. Moreover, none of the included studies evaluated patients’ perceptions and the impact of treatment on their OHRQoL. There are several fixed or removable devices used to correct anterior dental crossbite. The choice of a particular type of treatment depends on a close examination of various factors, such as the severity of malocclusion, the patient’s tolerance of discomfort caused by the treatment, and the professional skill of the orthodontist performing the treatment. Therefore, future research should be conducted addressing the impact of different early treatment protocols for anterior dental crossbite. Evidence-based dentistry, in the last 20 years, has been understood as the standard for oral health care worldwide.^30^ Clinicians should consider in their clinical routine both clinical experience and the best available evidence. However, awareness of patients’ needs and preferences is also an important component of orthodontic practice. The psychosocial characteristics of individuals along with their perceptions, expectations and values need to be taken into account when practitioners are providing orthodontic treatment.^31^

## CONCLUSION

While the quality of life of children undergoing treatment with upper removable appliance with digital springs improved, no change was observed in the quality of life of children submitted to treatment with resin-reinforced glass ionomer cement bite pads. This difference regarding the impact on OHRQoL, however, is unrelated to the protocol used.
